# Editorial: The Role of Myeloid-Derived Cells in the Progression of Liver Disease

**DOI:** 10.3389/fimmu.2019.02208

**Published:** 2019-09-18

**Authors:** Hannelie Korf, Reiner Wiest, Rajiv Jalan, Schalk van der Merwe

**Affiliations:** ^1^Laboratory of Hepatology, CHROMETA Department, KU Leuven, Leuven, Belgium; ^2^Maurice Müller Laboratories, Department for Biomedical Research, University of Bern, Bern, Switzerland; ^3^Department of Visceral Surgery and Medicine, Bern University Hospital, University of Bern, Bern, Switzerland; ^4^Liver Failure Group, Institute for Liver Disease Health, University College London, London, United Kingdom; ^5^Department of Gastroenterology and Hepatology, UZ Leuven, Leuven, Belgium

**Keywords:** liver disease, innate immunity, myeloid-derived cells, inflammation, macrophage

The liver is strategically positioned to drain the intra-abdominal organs through the portal vein. As such, the liver is constantly challenged with foreign food antigens, bacterial products that require a high degree of tolerance and restraint. In chronic liver disease, when hepatocyte damage occurs, this tolerant state is often lost resulting in local and systemic inflammation and fibrosis development, which may be detrimental to the host. It is well-established that macrophages, as highly diverse immune cells, play a central role in both the initiation as well as restoration of inflammation and fibrogenesis. Macrophages can be either embryonically seeded in organs where they are maintained through self-renewal (1) or derived from infiltrating bone marrow monocyte precursors (2, 3). Regardless of their origin, they adapt to microenvironmental cues within the niche they reside in and become imprinted with a unique transcriptional signature (4, 5). Technologies such as single-cell RNA sequencing accelerated discoveries in the field by uncovering the diversity of monocyte/macrophage functions even during steady-state conditions (6). Nevertheless, our understanding of the molecular mechanisms that goes astray during disease states, resulting in the loss of macrophages' ability to maintain homeostatic functions, remains incomplete. In this special Research Topic, experts in the field dissect the landscape of myeloid derived cells as well as neutrophils during chronic liver diseases. Understanding how myeloid cells contribute to injury and repair will enable the design of new therapies.

## Innate Immune Cell Crosstalk to Govern Inflammation and Repair During Acute and Chronic Liver Injury

In this Research Topic, Weston et al. provide a comprehensive overview regarding the role for myeloid-derived cells during acute and chronic hepatic injury. More specifically, they describe how tissue-resident Kupffer cells, infiltrating monocytes/macrophages, dendritic cells, and neutrophils act in concert to initiate an inflammatory response but also to regenerate tissue following injury. Finally, they touch upon the idea that strategies targeting liver macrophages would require precision medicine to specifically target pathogenic subsets only.

## Outlining the Extent of Innate Immune Defects During Cirrhosis Development and Acute-on-chronic Liver Failure

Patients with acute decompensated cirrhosis and acute-on-chronic liver failure (ACLF), display evidence of hepatic and systemic inflammation but paradoxically also features of immunosuppression, rendering them highly susceptible to infections. Within this Research Topic, a clinical investigation by Trebicka et al. demonstrated that the extent of systemic immune inflammation in acute decompensated cirrhotics correlates with a higher risk of disease progression toward ACLF or death of the patient. Another study by Alvarez-Silva et al. demonstrated that the levels of inflammatory cytokines and microbial richness are significantly higher in ascites fluid compared to plasma samples from patients with decompensated cirrhosis. However, the authors could not find any correlation between the bacterial DNA abundance and/or richness and the extent of systemic inflammation in these patients.

Providing some insight into the molecular mechanisms, Irvine et al. and Martin-Mateos et al. review the respective innate immune dysfuntions observed in cirrhosis and ACLF, within this Research Topic. Furthermore, Irvine et al. propose mechanisms that improve susceptibility to infections, while Martin-Mateos et al. highlights mechanisms targeting the gut, -high-grade systemic inflammation and reverting immune paralysis as therapeutic opportunities to improve clinical outcome of these patients. Also within this Research Topic, Triantafyllou et al. provide an overview of the role of monocytes/macrophages in driving systemic immunosuppression and hepatic inflammation thereby contributing to the pathophysiology of acute decompensated cirrhosis as well as ACLF. Additionally the authors describe the opportunities and challenges of therapeutic strategies aiming at reverting Kupffer cell activation, hampering monocyte recruitment to the liver or manipulating macrophage polarization to interfere in disease progression. Along the same line of investigation, Riva and Mehta provide evidence for epigenetic mechanisms, and the role of checkpoint receptors in regulating monocyte function. In combination with recent advances in the field whereby metabolic rewiring can influence immunological functions (7), these studies open up new opportunities of targetable pathways that may be exploited to improve monocyte function in ACLF.

Finally, Moreau et al. placed the spotlight on dysfunctional neutrophils during cirrhosis within the Research Topic. More particularly, they provide an overview of defects in intracellular signaling pathways, impaired activation of the NADPH oxidase complex, myeloperoxidase (MPO) release and defective bactericidal activity within neutrophils during cirrhosis. Importantly, they report some studies that suggest that defective neutrophil functions, at least *in vitro*, could be rescued by TLR7/8 agonists.

## Taking the Break off Monocytes, Drives Inflammation, and Intestinal Barrier Breach

Patients with advanced stages of cirrhosis often exhibit a dysfunctional intestinal barrier, whereby luminal bacteria and their products translocate into the circulation and reach the liver via the portal vein (gut-liver axis). Very exciting recent research elucidate for the first time the mechanisms of intestinal barrier breach during cirrhosis (8). Nevertheless, how exactly monocytes/macrophages contribute to intestinal barrier dysfunction remains incompletely understood. Although not directly linked to an experimental liver disease model, an interesting study by Mouhadeb et al., may shed some light on this aspect by demonstrating that disruption of COMMD10, a protein with yet unknown function, unleashes the inflammatory capacity of circulating Ly6C^hi^ monocytes, resulting in intestinal barrier dysfunction and elevated bacterial translocation to the mouse liver. It would be intriguing to verify whether similar mechanisms set the stage for pathogenic bacterial translocation during cirrhosis development.

## Targeting Macrophages During Non-alcoholic Fatty Liver Disease

Non-alcoholic fatty liver disease (NAFLD) is hallmarked by chronic low-grade inflammation and lipid accumulation in the liver as well as in extra-hepatic sites such as adipose tissue. Furthermore, a complex inter-organ crosstalk fuels the onset and progression of hepatic injury and fibrosis development. Within this disease setting, it is well-established that myeloid-derived cells play a prominent role in regulating inflammation and metabolism. In this Research Topic, Hundertmark et al. provide a comprehensive overview of the functional and phenotypic versatility of myeloid cells as well as the microenvironmental signals that trigger their activation during NAFLD progression. Additionally, they point out the existence of an orchestrated interplay between myeloid cells in different compartments, such as circulation, gut, adipose tissue, and the liver. Extending along the same line of investigation, Vonghia et al. review current data on experimental NAFLD treatment strategies whereby myeloid-derived cells constitute the targeted population.

In another manuscript within this Research Topic, Liao et al. demonstrated that palmitate-induced hepatocyte stress resulted in the release of extracellular vesicles enriched in the lipotoxic molecule, sphingosine 1-phosphate (S1P). These extracellular vesicles promoted macrophage chemotaxis through interaction with the S1P1 receptor on the surface of the macrophages. Although awaiting further *in vivo* confirmation, the authors suggest this to be a novel signaling axis for macrophage recruitment during NAFLD, where hepatic lipotoxicity prevails.

## Macrophages Going Off Track in the Aging Liver

The process of aging is closely associated with a number of degenerative modifications in the liver, where hepatic structure and cell function tend to decline. In this Research Topic, Stahl et al. review macrophage deficits in mitochondrial function,—decline in autophagy and altered proinflammatory function, are discussed as possible mechanisms that may be relevant during age-related liver diseases.

## Neutrophils Fueling Liver Ischemia and Reperfusion Injury

Hepatic ischemia/reperfusion (I/R) is an important cause of liver damage occurring during hepatic resection and liver transplantation. Neutrophils have been shown to be one of the cellular players contributing towards tissue injury, however the molecular mechanisms involved have not been completely defined. Within this Research Topic, Sun et al. implicate that MAPK-activated protein kinase 2 (MK2) contribute to hepatic I/R since its ablation protects against hepatic I/R injury in a murine model. This result implicates MK2 as a potential novel therapeutic target for I/R injury.

## Conclusions

Emerging evidence demonstrated that myeloid-derived cells play a key role in the initiation, and progression of liver disease. This Research Topic provides multiple examples of how different myeloid cell subsets but also neutrophils can contribute to the inflammatory processes that underlie the clinical manifestations of various liver diseases. Such a compilation of relevant information may uncover new therapeutic targets and ultimately lead to improved outcomes in patients with advanced liver disease.

## Author Contributions

All authors listed have made a substantial, direct and intellectual contribution to the work, and approved it for publication.

### Conflict of Interest Statement

The authors declare that the research was conducted in the absence of any commercial or financial relationships that could be construed as a potential conflict of interest.
